# Short-Term Workload Patterns Before Achilles Tendon Rupture in National Basketball Association Players: A Descriptive Epidemiology Study

**DOI:** 10.1177/23259671261463385

**Published:** 2026-07-24

**Authors:** Vineet Kumar, Luke Sang, Wyatt David, Nirav Pandya

**Affiliations:** * Department of Orthopaedic Surgery, University of California San Francisco, San Francisco, California, USA; † School of Medicine, University of California San Francisco, San Francisco, California, USA; Investigation performed at the Department of Orthopaedic Surgery, University of California San Francisco, San Francisco, California, USA

**Keywords:** Achilles tendon ruptures (ATR), workload, Player Efficiency Rating (PER), early sports specialization, schedule congestion

## Abstract

**Background:**

Achilles tendon rupture (ATR) is one of the most serious injuries in professional basketball, requiring nearly a year of recovery and resulting in declines in player performance and career longevity. In the 2024-2025 NBA season, a record-high 7 ATRs highlighted the need to evaluate workload-related risk factors.

**Hypothesis:**

NBA players experiencing ATR may have a higher-than-average workload in games preceding injury, which could be associated with increased risk.

**Study Design:**

Descriptive epidemiology study.

**Methods:**

This retrospective exploratory review analyzed publicly available NBA data from the 2014-2015 to 2024-2025 seasons. Inclusion required players to have sustained a basketball-related ATR during a regular season or playoff game after participating in at least 1 full game that season. Variables included age, position, specialization, meters run, minutes played (5-, 10-, 15-game spans before ATR), games played, and scheduling metrics (back-to-backs, rest days). Data sources included official NBA statistics, StatMuse, and ESPN. Statistical analyses included paired and unpaired *t* tests, 1-way analysis of variance, and Spearman correlations.

**Results:**

Nineteen players (19 ATRs; mean age, 27.8 ± 3.2 years; mean league experience: 7.4 ± 3.2 years) sustained basketball-related ATRs. Playing time was significantly higher in the 10 games (29.0 ± 2.1 vs 27.4 ± 2.0 min/game; *P* = .01) and 15 games (28.6 ± 2.1 vs 27.4 ± 2.0 min/game; *P* = .03) preceding injury compared with each player’s season average. Years of league experience were positively correlated with workload measures, including total season minutes (*P* = .02) and minutes in the 5 games preceding rupture (*P* = .0498), such that veteran players generally accumulated higher workload, whereas ATRs among less-experienced players occurred in the setting of comparatively lower observed workload.

**Conclusion:**

Significant workload spikes were found in the 10 to 15 games preceding Achilles ruptures for NBA players. Veteran players carried heavier cumulative workloads, whereas younger players ruptured tendons under lighter use. Proactive workload management, athlete education, and targeted prevention strategies may help preserve player health and career longevity in the NBA.

Achilles tendon rupture (ATR) is a severe soft tissue injury among athletes in the National Basketball Association (NBA), with important implications for return to play, performance, and career longevity.^4^ Over the past 35 years, ATRs have remained a persistent concern in professional basketball, with the NBA averaging approximately 1.3 ruptures per season between 1990 and 2023.^8^ However, during the 2024-2025 season, 7 players sustained an ATR during the regular season or playoffs, representing an increase compared with historical observations, in which the maximum number of in-season ruptures in a single year was 4.^13^ The injury is associated with prolonged recovery, with NBA players requiring an average of approximately 10.5 months to return to play, frequently resulting in the loss of the current season and much of the following season.^7^ Even among players who successfully return to play, postinjury declines in on-court performance have been reported, including reductions in Player Efficiency Rating (PER), along with decreased postinjury career longevity.^3,10^ Multiple factors have been discussed in relation to ATR in professional basketball, including cumulative playing exposure, minutes played, and the substantial physical demands of competition.^1^

In recent years, increasing attention has focused on challenges related to workload accumulation and schedule congestion in the NBA.^11,17^ Players and coaches have raised concerns regarding back-to-back games and compressed scheduling, citing increased physical stress and limited recovery time.^12^ These concerns have become more prominent as teams experience extended stretches of games within short time intervals, raising questions regarding the balance between player health and performance demands. Concurrently, efforts to better characterize ATR mechanisms in the NBA have expanded, including the application of advanced analytics and artificial intelligence–based tools to explore potential injury risk factors.^13^

Despite the clinical significance of ATR and increasing awareness of its burden in the NBA, factors underlying recent increases in ATR incidence remain incompletely understood.^6,16^ In particular, short-term workload patterns preceding injury have not been well characterized in professional basketball, despite ongoing concerns regarding cumulative playing demands and limited recovery opportunities during the NBA season. Although workload management strategies have become more prevalent across the league, objective data describing workload fluctuations immediately before ATR remain limited.

The purpose of this study was to explore workload-related factors associated with ATR in NBA players. By examining advanced game statistics in the seasons leading up to injury, this study aims to identify patterns in short-term workload fluctuations observed before ATR. We hypothesize that NBA players experiencing ATR may have a higher-than-average workload in games preceding injury, which may be associated with increased risk.

## Methods

This was an exploratory retrospective review of the official NBA advanced statistics database and publicly available information on NBA players from the 2014-2015 season through the 2024-2025 season. Given the multifactorial nature of this serious injury, the objective of this study was to evaluate workload-related variables that may contribute to ATRs in NBA players. Players from any team who tore their Achilles tendon between 2014 and 2025 were identified (ESPN, The Athletic, Yahoo Sports). Inclusion criteria required that the injury occurred during a professional-level basketball activity in a regular season or playoff game after the player had participated in at least 1 full game for their respective team. Exclusion criteria included non–basketball related ATRs, off-season injuries, revision or recurrent ruptures, and players who had participated in <1 full game before injury.

A total of 25 players were identified as having torn their Achilles since the 2014-2015 season; however, 6 were excluded due to non–basketball related injuries, off-season injuries, or an insufficient sample size of games played (1 full game) before their injury. Our final sample included 19 NBA players.

Our dataset included a variety of variables: player position, age, history of early sport specialization (single-sport vs multisport participation), average meters run during the injury season versus average career meterage (excluding the injury season), total meterage run during the injury season versus total career meterage (excluding the injury season), minutes played in the preceding span of 5, 10, and 15 games, season average minutes per game calculated for each player in their injury season, excluding the injury game, average career minutes per game in seasons before injury, number of games played in the 30 days before injury, number of back-to-back games in the past 30 days, and total days off in the 30 days preceding injury. Game-based preinjury windows were selected rather than week-based windows to standardize the number of competitive exposures across players, given the variable game frequency in NBA scheduling.

To gather information on high school multisport participation, we used publicly available sources (MaxPreps, Hudl, ESPN) to identify which NBA players had competed in sports other than basketball. To be classified as a multisport athlete, players had to have participated in another sport during high school. Sports played before high school were not considered. For international players, multisport participation was evaluated among players aged 14 to 18. We conducted searches across all publicly accessible sources, including player interviews, biographies, news articles, and high school sports databases. Given the reliance on publicly available high school participation records and the inability to assess sport involvement before age 14, early sport specialization was evaluated as an exploratory variable.

Four players in the final sample sustained the injury during the playoffs. Workload data regarding the number of meters run per game and per season were obtained from the official NBA advanced statistics database (www.nba.com/stats). Meterage was defined as the total distance run during a game, measured using player-tracking technology, which was standardized league-wide beginning in the 2013-2014 season. For players who played in seasons before 2013-2014, career meterage was included from 2013 through the season before their ATR. For season-specific workload, we used only regular-season meterage to calculate averages for the season injured, unless the participant was hurt in the playoffs. Regarding total career meterage, both regular-season and playoff data were included when applicable to reflect the complete meterage load over a player’s career.

Information on player age at the time of injury and minutes played during the season and previous seasons was acquired from StatMuse (https://www.statmuse.com/), an artificial intelligence company specializing in sports statistics. To determine the number of games played in the 30 days before injury, we used StatMuse to review the preceding month’s games and cross-checked them with the official NBA calendar for that year to ensure accuracy. For minute-based variables such as average minutes per season or in prior seasons, regular-season minutes were used in all averaged calculations to maintain consistency. Preceding game stats (minute-based variables) do not include the injured game and are based on the games on the player’s schedule preceding the Achilles tear. Playoff minutes were only considered if an injury occurred in the playoffs.

All injuries were verified through a combination of official team statements and public reporting via ESPN.com and other reputable sports journalism sources. Additional confirmation of games played, minutes per season, game spans, and schedule congestion was done using ESPN (www.espn.com).

### Statistical Analysis

Descriptive statistics were calculated to characterize the player cohort. Unpaired *t* tests and 1-way analysis of variance were used, when appropriate, to determine differences in age at Achilles tear and schedule congestion leading up to the injury between player position (guard vs forward vs center vs combo), starter status (starter vs bench), and early sport specialization status (single vs multisport). Paired *t* tests were used to determine significant differences in players’ workload during the season of their Achilles tear, compared with the rest of their career preceding the injury (distance traveled and minutes played), and workload in games leading up to their Achilles tear, compared with the rest of that season. Spearman correlations were also calculated to evaluate the relationships between player workload in their Achilles tear season and age and years in the NBA. Given the small sample size and descriptive study design, all analyses are considered exploratory and hypothesis-generating. Thus, no corrections for multiple comparisons were applied. Statistical significance at *P* < .05 is reported as a descriptive threshold only. All analyses were performed using Stata 19.0 (StataCorp LLC).

## Results

A total of 19 NBA players met the inclusion criteria for this study. The mean age was 27.8 ± 3.2 years (Table 1), and most players played either guard (42.1%) or multiple positions (36.8%). Almost all players who tore their Achilles played only basketball in high school (89.5%), and the 2024-2025 NBA season saw the highest number of Achilles tear injuries (31.6%). Three players in the cohort of 19 (15.8%) had a calf injury within 15 games of their Achilles tear, whereas 8 players had no injuries documented (42.1%).

**Table 1:** Cohort Characteristics 2014-2025 (n = 19)^*a*^

Characteristic	Value
Age, y	27.8 ± 3.2 (22-34)
Experience, y	7.4 ± 3.2 (3-13)
Position	Guard: 8 (42.1) Forward: 2 (10.5) Center: 2 (10.5) Combo: 7 (36.8)
Team role	Starter: 12 (63.2) Bench: 7 (36.8)
Early sports specialization status	Single sport (89.5) Multisport (10.5)
Season of ATR	2014-2015: 4 (21.1) 2015-2016: 1 (5.3) 2016-2017: 1 (5.3) 2017-2018: 1 (5.3) 2018-2019: 2 (10.5) 2019-2020: 3 (15.8) 2020-2021: 0 (0.0) 2021-2022: 0 (0.0) 2022-2023: 1 (5.3) 2023-2024: 0 (0.0) 2024-2025: 6 (31.6)
Injury within 15 games of ATR	Ankle: 1 (5.3) Calf: 3 (15.8) Groin: 1 (5.3) Hip: 1 (5.3) Knee: 1 (5.3) Other: 4 (21.1) None: 8 (42.1)
Schedule congestion: games played within 30 days of ATR	9.9 ± 4.1 (1-16)
Schedule congestion: B2B within 30 days of ATR	0: 5 (26.3) 1: 3 (15.8) 2: 7 (36.8) 3: 4 (21.1)

a Data are reported as mean ± SD (range) or n (%). ATR, Achilles tendon rupture; B2B, back-to-back games.

There were no differences in the age at which players tore their Achilles across guards, forwards, centers, and combo-position players (*P* = .71) (Table 2). Further, we noted no differences in schedule congestion when looking at B2Bs or games played within 30 days of the Achilles tear across these positions (*P* = .15; *P* = .34). When comparing starter versus bench players and single versus multisport early sport athletes, we found no significant differences across age and scheduling metrics.

**Table 2:** Comparison of Age at Achilles Tear and Schedule Congestion Across Player Characteristics^*a*^

Group	Subgroup	Age at ATR, y	B2Bs Within 30 Days	Games Within 30 Days	*P*, Age/B2B/Games
Position	Guard	29.5 ± 3.5	1.6 ± 1.1	10.6 ± 3.8	.71/.15/.34
	Forward	28.5 ± 2.1	0.0 ± 0.0	10.5 ± 4.9	
	Center	29.5 ± 3.5	2.5 ± 0.7	13.5 ± 0.7	
	Combo	26.7 ± 2.5	1.6 ± 1.1	7.9 ± 4.5	
Playing status	Starter	28.4 ± 2.7	1.5 ± 0.4	10.1 ± 1.4	.28/.90/.80
	Bench	26.7 ± 4.0	1.6 ± 0.2	9.6 ± 1.0	
Early sports specialization status	Single-sport	27.0 ± 1.0	1.5 ± 0.3	9.8 ± 1.1	.73/.97/.70
	Multisport	27.9 ± 0.8	1.5 ± 0.5	11.0 ± 0.0	

a Data are reported as mean ± SD. ATR, Achilles tendon rupture; B2B, back-to-back games.

We noted a significant increase in average minutes played in both the 10 (29.0 ± 2.1 min/g [95% CI, 24.5-33.5] vs 27.4 ± 2.0 min/g [95% CI, 23.2-31.6]; *P* = .01) and 15 (28.6 ± 2.1 min/g [95% CI, 24.2-33.0] vs 27.4 ± 2.0 min/g [95% CI:,23.2-31.6]; *P* = .03) games preceding a player’s Achilles tear compared with the rest of their season (Table 3). We found no differences in average distance run (*P* = .91) or average minutes played (*P* = .93) in the season of a player’s Achilles tear compared to the rest of their career preceding that injury season.

**Table 3:** Comparison of Players’ Workload During Achilles Tear Season and Games Leading Up to Injury^*a*^

	Average Distance Run, m/g	95% CI	*P*
Achilles tear season	3094.3 ± 210.2	2852.5-3536.0	.91
Career preceding Achilles tear season	3109.6 ± 187.3	2716.1-3506.0	
	Average Minutes, min/g		
Achilles tear season	26.8 ± 2.0	22.7-31.0	.93
Career preceding Achilles tear season	26.7 ± 1.8	22.9-30.6	
	Average Minutes, min/g		
Achilles tear season	26.8 ± 2.0	22.7-31.0	.07
Preceding 5 games to Achilles tear	28.2 ± 2.0	24.0-32.5	
	Average Minutes, min/g		
Achilles tear season	**27.4 ± 2.0**	**23.2-31.6**	.**01**
Preceding 10 games to Achilles tear^*b*^	**29.0 ± 2.1**	**24.5-33.5**	
	Average Minutes, min/g		
Achilles tear season	**27.4 ± 2.0**	**23.2-31.6**	.**03**
Preceding 15 games to Achilles tear^*b*^	**28.6 ± 2.1**	**24.2-33.0**	

a Data are reported as mean ± SD. Statistically significant findings are shown in bold.

b One player excluded due to insufficient games.

Further, a positive correlation was found between a player’s years in the NBA and minutes played in their Achilles tear season (*P* = .02) (Table 4). There was also a positive correlation between years in the NBA and the average minutes played in the 5 games preceding an Achilles tear (*P* = .0498). No significant correlations were found between a player’s age at the time of their Achilles tear and workload metrics.

**Table 4:** Correlations of Players’ Age and Year in the NBA With Workload in Achilles Tear Season^*a*^

Variable	Age	Years in League
Average distance run (m/g) in ATR season	0.33 (.16)	0.45 (.05)
Average minutes (min/g) in ATR season	0.36 (.13)	**0.53 (.02)**
Average minutes (min/g) in preceding 5 games	0.32 (.18)	**0.46 (.0498)**
Average minutes (min/g) in preceding 10 games^*b*^	0.21 (.39)	0.40 (.10)
Average minutes (min/g) in preceding 15 games^*b*^	0.26 (.29)	0.43 (.07)

a Data are presented as Spearman rho (*P* value). Statistically significant findings are shown in bold. ATR, Achilles tendon rupture.

b One player excluded due to insufficient games.

## Discussion

The major findings in this descriptive case series of NBA players who sustained ATRs were related to workload patterns preceding injury. Players demonstrated increased minutes played during the 10-game (29.0 ± 2.1 min/g [95% CI, 24.5-33.5] vs 27.4 ± 2.0 min/g [95% CI, 23.2-31.6]; *P* = .01) and 15-game (28.6 ± 2.1 min/g [95% CI, 24.2-33.0] vs 27.4 ± 2.0 min/g [95% CI, 23.2-31.6]; *P* = .03) spans preceding injury, indicating that short-term elevations in playing time commonly occurred in the period immediately before ATR. In contrast, no significant differences were identified across age, player position, role, early sport specialization, or measures of schedule congestion, suggesting that these injuries were not confined to specific subgroups. Years of NBA experience were positively correlated with cumulative minutes played during the injury season, such that veteran players generally carried higher playing demands, whereas ATRs among less-experienced players occurred in the setting of comparatively lower observed workload. Together, these findings suggest that although workload metrics alone do not fully characterize ATR occurrence, they may provide useful descriptive context when considered alongside cumulative exposure and individual susceptibility. Importantly, these results extend the existing literature by offering temporally focused, hypothesis-generating observations of workload patterns immediately preceding injury in NBA athletes.

The observation of increased minutes played in the 10 to 15 games preceding ATR adds temporal specificity to existing literature on workload and injury in professional basketball. Prior work has identified player minutes as an important factor associated with season-ending injuries, and the present findings build on this literature by characterizing short-term workload patterns immediately preceding ATR.^9^ Although season-long workload measures did not differ substantially from historical baselines, short-term fluctuations in playing time were observed in the period leading up to injury. These findings do not establish workload as a risk factor for ATR but rather describe workload patterns temporally associated with injury occurrence within the injured cohort.

An additional observation concerned the relationship between NBA experience and cumulative workload within the injured cohort. Players with greater NBA experience accumulated higher total minutes during the injury season, whereas ATRs among less-experienced players occurred in the setting of comparatively lower observed workload. This represents a potentially important descriptive pattern and may reflect underlying differences in factors such as prior exposure history, biomechanical characteristics, or foundational conditioning. However, these explanations are speculative and cannot be evaluated within the present dataset. These findings should not be interpreted as indicating differential risk by experience level but instead reflect heterogeneity in workload exposure and injury context among NBA athletes. Prior epidemiologic work has reported that a substantial proportion of NBA ATRs occur early in the season, which is directionally consistent with the present cohort, in which several injuries occurred during the early regular season or shortly after brief periods of missed play.^1,7^ Nonetheless, the present findings remain descriptive and do not establish mechanistic or temporal causation.

No significant associations were identified between ATR occurrence and age, player position, role, early sport specialization, or measures of schedule congestion. Early sport specialization was examined strictly as an exploratory variable, as classification was limited to multisport participation during high school and did not capture sports involvement before age 14. Therefore, findings related to this variable should be interpreted with caution. Collectively, these results suggest that ATRs in NBA players are not confined to specific demographic or positional subgroups. Additionally, despite widespread concern regarding NBA scheduling demands, schedule congestion measures were not associated with ATR in this cohort, which may reflect recent league-wide efforts to reduce back-to-back games and overall schedule density, as well as substantial variability in individual playing roles and exposure across seasons. Although prior literature has suggested that multisport participation may be associated with greater tolerance to workload, such interpretations remain speculative in the context of the present study.^2,5,14,15^ The observed workload patterns, including short-term increases in minutes preceding injury and heterogeneity across levels of experience, underscore the multifactorial nature of ATR in professional basketball.

When considered in the broader context of professional basketball, the present findings are consistent with prior epidemiologic observations that ATR occurs across a wide range of players and competitive settings. Injuries in this cohort occurred during both the regular season and postseason, and no significant associations were identified with measures of schedule congestion or player role. These observations suggest that ATRs in NBA athletes may arise under diverse competitive conditions rather than being driven by a single scheduling or exposure pattern. The occurrence of several injuries early in the season further highlights the potential relevance of recent workload context, although definitive conclusions regarding timing or mechanism cannot be drawn from the present data. Collectively, these findings reinforce the view of ATR as a multifactorial injury shaped by heterogeneous exposures and individual factors rather than a uniform risk profile.^1,6,7^

Interpretation of these findings should be considered in the context of several important data limitations. Publicly available datasets do not capture key components of workload and injury context, including explosive movement frequency, chronic tendinopathy history, practice loads, prior workload trends, and team-level load-management decisions. In addition, objective measures of game intensity, particularly relevant for postseason injuries, were not available. The absence of these variables limits the ability to fully characterize workload exposure preceding ATR and reinforces the descriptive and exploratory nature of the present analysis. These limitations are detailed further below.

Several limitations of this study should be acknowledged. Comprehensive career meterage data were unavailable for players whose NBA careers began before the 2013-2014 season, resulting in the exclusion of 8 players and limiting the assessment of long-term cumulative workload preceding ATR. Although total minutes played were available, the absence of detailed meterage data from earlier seasons constrained granular workload evaluation, particularly for players with extensive careers. Variability in NBA scheduling across seasons is an important consideration, as differences in back-to-backs, travel, rest, and recent league efforts to reduce schedule congestion have altered exposure patterns. This was evident in subgroup analyses, particularly among “Combo” players, who showed wide variability in games played in the 30 days before injury (7.9 ± 4.5), complicating comparisons across positions and seasons. Despite spanning over 10 NBA seasons, the final analytic cohort included only 19 players, limiting statistical power and increasing the risk of Type I and II errors. No correction for multiple comparisons was applied, consistent with the exploratory and hypothesis-generating intent of this study. Accordingly, reported *P* values should be interpreted as descriptive and not inferential. Additionally, although the paired *t* test approach compares player-level summary means, the preinjury window games are included in the full season average used as the comparator, as game-level separation was not feasible with publicly available data. Future work with larger cohorts and game-level longitudinal data, using mixed-effects models, is needed to further characterize the workload trajectories preceding injury. Interpretation is further limited by the absence of a matched healthy control group. A within-subject design was therefore employed, using each player’s historical workload as an internal comparator. Although this approach reduces interplayer variability, it cannot account for league-wide workload norms and does not permit direct comparison with uninjured players. Workload comparisons were conducted using game-based windows rather than week-based rolling averages, as NBA scheduling variability would produce inconsistent exposure counts across players when using time-based windows, and because of a lack of publicly available data. Future studies should consider computing formal ratios of acute to chronic workload using week-based rolling windows if such data become available.

Finally, several key workload-related variables were unavailable in public datasets, including game intensity, explosive movement frequency, chronic tendinopathy history, practice loads, prior workload trends, and team-level load-management decisions. Although 4 ATRs occurred during the playoffs, when demands are higher, game intensity could not be quantified, and playoff-specific analyses were not feasible due to the small sample size and lack of objective measures of game intensity in publicly accessible datasets. These limitations restrict full characterization of workload exposure and reinforce the exploratory nature of the study. Despite these limitations, this study provides descriptive insight into short-term workload patterns preceding ATR in NBA players. Future studies with larger cohorts, matched controls, objective intensity metrics, and comprehensive longitudinal workload data are needed to better define the relationship between workload dynamics and ATR risk in professional basketball.

## Conclusion

Our study demonstrated significant workload spikes in the 10 to 15 games preceding Achilles ruptures for NBA players. Veteran players carried heavier cumulative workloads, whereas younger players ruptured tendons under lighter usage. These exploratory findings align with prior literature suggesting that both sudden increases in playing time and cumulative career demands can compromise tendon durability, and such findings warrant further investigation. Proactive workload management, athlete education, and targeted prevention strategies may help preserve player health and career longevity in the NBA.
